# Ceftriaxone-induced hemolytic anemia with severe renal failure: a case report and review of literature

**DOI:** 10.1186/s40360-018-0257-7

**Published:** 2018-10-25

**Authors:** Hans Benno Leicht, Elke Weinig, Beate Mayer, Johannes Viebahn, Andreas Geier, Monika Rau

**Affiliations:** 1 Department of Internal Medicine II, University Hospital Würzburg, Oberdürrbacherstraße 6, 97080 Würzburg, Germany; 2 Institute of Transfusion Medicine and Haemotherapy, University of Wuerzburg, Wuerzburg, Germany; 3 Institute of Transfusion Medicine, Charité - Universitätsmedizin Berlin, corporate member of Freie Universität Berlin, Humboldt-Universität zu Berlin, and Berlin Institute of Health, Berlin, Germany

**Keywords:** Drug-induced immune hemolytic anemia, Ceftriaxone, Hemolysis

## Abstract

**Background:**

Drug induced immune hemolytic anemia (DIIHA) is a rare complication and often underdiagnosed. DIIHA is frequently associated with a bad outcome, including organ failure and even death. For the last decades, ceftriaxone has been one of the most common drugs causing DIIHA, and ceftriaxone-induced immune hemolytic anemia (IHA) has especially been reported to cause severe complications and fatal outcomes.

**Case presentation:**

A 76-year-old male patient was treated with ceftriaxone for cholangitis. Short time after antibiotic exposure the patient was referred to intensive care unit due to cardiopulmonary instability. Hemolysis was observed on laboratory testing and the patient developed severe renal failure with a need for hemodialysis for 2 weeks. Medical history revealed that the patient had been previously exposed to ceftriaxone less than 3 weeks before with subsequent hemolytic reaction. Further causes for hemolytic anemia were excluded and drug-induced immune hemolytic (DIIHA) anemia to ceftriaxone could be confirmed.

**Conclusions:**

The case demonstrates the severity of ceftriaxone-induced immune hemolytic anemia, a rare, but immediately life-threatening condition of a frequently used antibiotic in clinical practice. Early and correct diagnosis of DIIHA is crucial, as immediate withdrawal of the causative drug is essential for the patient prognosis. Thus, awareness for this complication must be raised among treating physicians.

## Background

Ceftriaxone is a broad-spectrum cephalosporin that is used for the treatment of diverse bacterial infections. It is known to cause hemolysis by inducing complement activating drug-dependent antibodies of mainly immunoglobulin M (IgM)-type, resulting in “immune-complex” type immune hemolytic anemia [1–3]. During the last years, ceftriaxone has been one of the most important drugs that were shown to be responsible for drug-induced immune hemolytic anemia (DIIHA) [3–6]. Ceftriaxone-induced immune hemolytic anemia (IHA) is characterized by sharp decrease of hemoglobin, a high rate of organ failure and a mortality of at least 30% [2, 3, 6–8], whereas children present with a more severe clinical picture and have a worse prognosis than adults [2, 5–7]. Here, we present the case of a 76-year-old patient with ceftriaxone-induced IHA who was treated in our department and could be managed to survive without persistent physical impairment. We give an overview of the pathophysiology and therapeutic options of DIIHA, a rare and probably underdiagnosed condition. As DIIHA is caused by frequently used medications like ceftriaxone, it is necessary to raise awareness of this immediately life-threatening condition among treating physicians. Antibiotic treatment should be strictly restricted to proper indications to prevent complications such as DIIHA [9].

## Case presentation

**Fig. 1:**
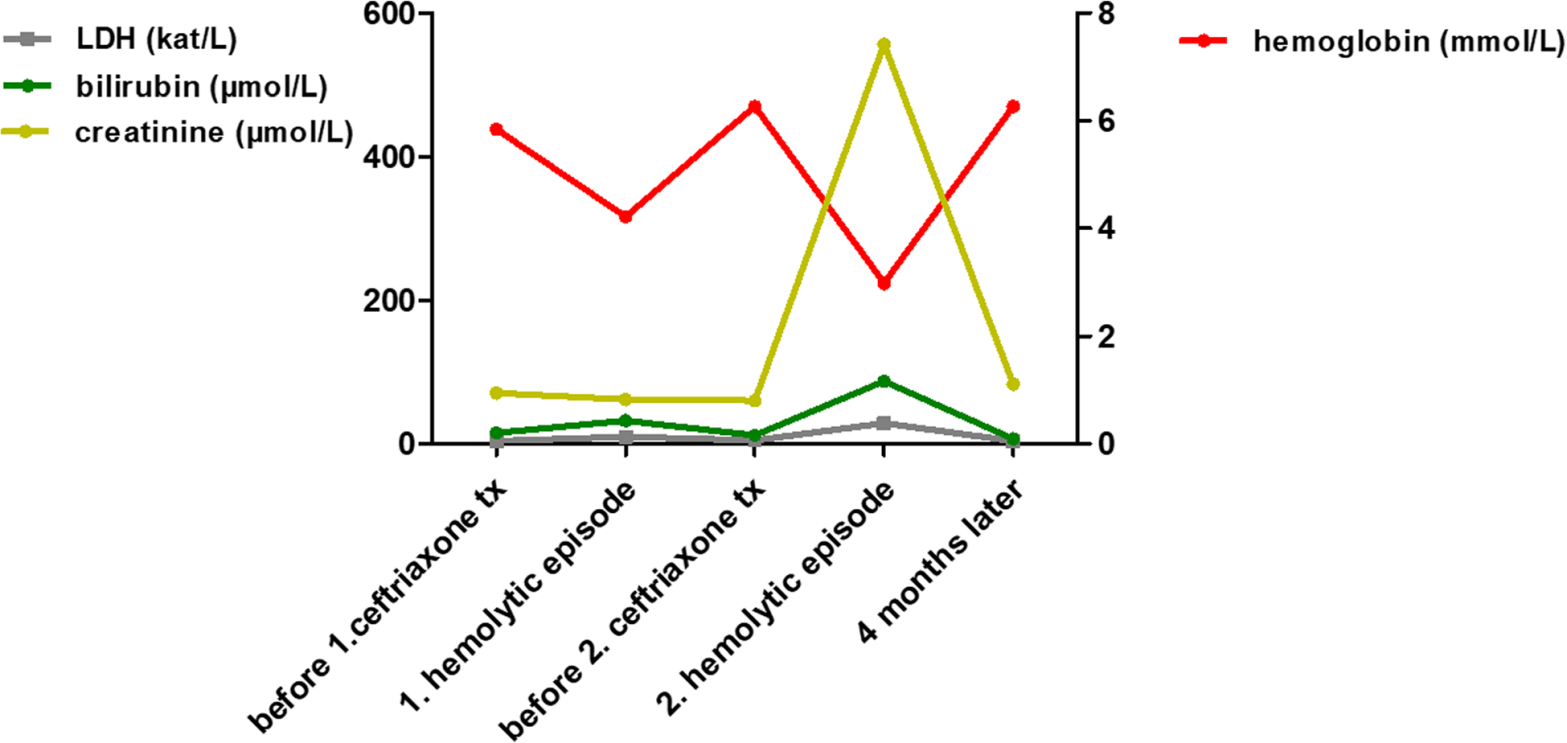
Representative laboratory parameters during disease course

In January 2017, a 76-year-old male patient was admitted to our hospital with ascites and dyspnea. In the patient’s history, a portal vein thrombosis was known for more than 10 years due to relapsing, necrotizing biliary pancreatitis. At that time a cholecystectomy with biliodigestive anastomosis was performed. Ascites was analysed after large-volume paracentesis without signs of spontaneous bacterial peritonitis or malignancy. On the second day after hospitalization, an esophagogastroduodenoscopy was performed to screen for esophageal varices. After the intervention, the patient developed fever and chills. Cholangitis was suspected due to biliodigestive anastomosis, increase of cholestasis parameters and an antibiotic treatment with ceftriaxone was started the same day (dose 4 g intravenously). Immediately after drug application the patient complained about nausea, vomited and developed dyspnea, confusion and a positive shock index (systolic RR < 100 mmHg, cardiac frequency 140 /min). The patient was referred to our intensive care unit and the antibiotic regime was escalated to piperacillin/tazobactam and ciprofloxacin for sepsis therapy. The patient received no further dose of ceftriaxone. Laboratory analysis about 1 h after application of ceftriaxone showed first signs of hemolysis with an elevated lactate dehydrogenase (LDH) (1,116 U/L (18.6 μkat/l); baseline 290 U/L (4.83 μkat/l)) and a decrease in hemoglobin (6.4 g/dl (3.97 mmol/l), baseline 8.5 g/dl (5.28 mmol/l)). Coagulation parameters were significantly disturbed indicating DIC with an international normalized ratio (INR) of 3.31 (baseline 1.29), fibrinogen not measurable, thrombocytopenia down to 56,000/μl (baseline 203,000/μl). During the next days, the patient developed an increase in leukocytes (up to 23,000/μL) and in infection parameters (peak C-reactive protein (CRP) 9.35 mg/dl (890.48 nmol/l), peak procalcitonin (PCT) 134 μg/l). Additionally, hemolysis aggravated with a nadir hemoglobin of 4.8 g/dl (2.98 mmol/l), an elevated LDH up to 1,734 U/L (28.9 μkat/l) and suppressed haptoglobin < 0.1 g/l. (course of laboratory parameters is depicted in Fig. 1). Furthermore, the patient subsequently developed a severe acute kidney failure with uremia (peak creatinine 6.29 mg/dl (556.04 μmol/l), urea 192.3 mg/dl (32.11 mmol/l)) and intermittent hemodialysis was necessary for 14 days. A kidney biopsy was performed and showed a severe acute tubular damage fitting with shock-induced injury and/or tubular-toxic effects of free hemoglobin/hemin.

**Fig. 2:**
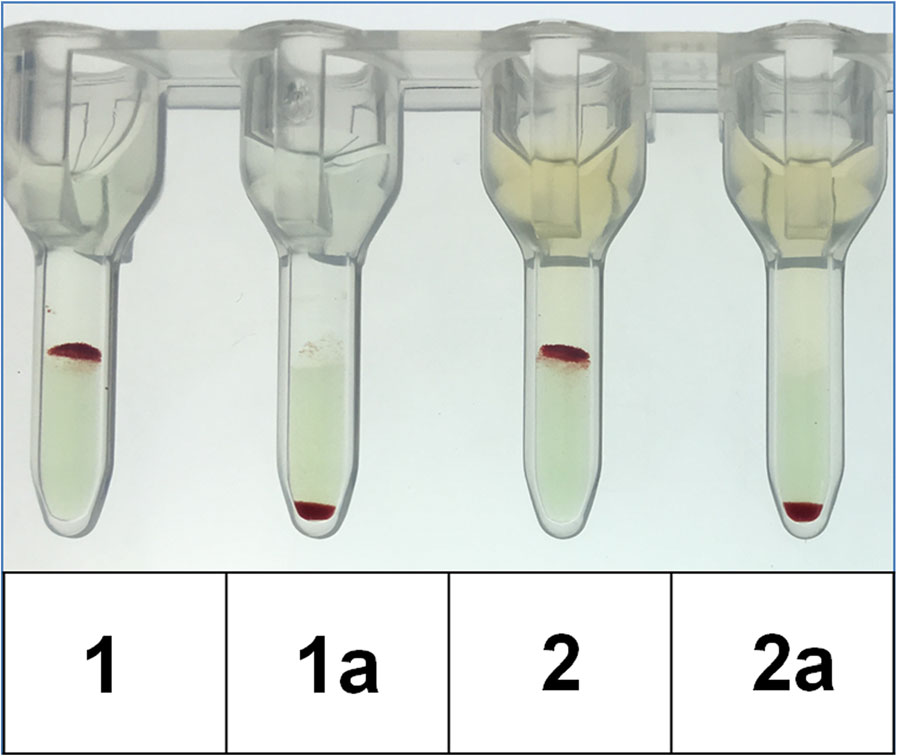
Serological investigation of ceftriaxon-dependent antibody using gel card technique (BioRad, Cressier sur Morat, Switzerland). Results showing strong agglutination (4+) of the patient’s plasma and eluate in the presence of the drug, but negative results without ceftriaxone added. Patient’s eluate (1) or plasma (2), ceftriaxone and untreated RBCs; Negative controls: patient’s elutate (1a) or plasma (2a), saline (instead of ceftriaxone) and untreated RBCs

The massive hemolytic reaction came suddenly and was unexpected. After exclusion of hematological comorbidities, a detailed patient history with current drug exposure was performed. Before admission to our department the patient had been hospitalized in our surgical department due to pneumothorax after pacemaker implantation. During this hospitalisation (< 3 weeks before the current admission) the patient had already been treated with ceftriaxone for at least 6 days and had already developed mild hemolysis in laboratory analysis without further consequences at that time. Further detailed diagnostic showed a positive Coombs’ direct antiglobulin test (DAT) for immunoglobulin M (IgM), immunoglobulin G (IgG) and complement factor C3d. On Naranjo Scale, a probability scale for adverse drug reactions, the patient would have reached a value of 9 points (maximal score 13 points, with values ≥9 points indicating a definite adverse drug reaction) [10]. The suspected DIIHA was proven by reference laboratory analysis (Institute of Transfusion Medicine, Charité, Berlin), confirming the presence of a strongly agglutinating ceftriaxone-dependent antibody (Fig. 2**).**

The patient’s situation stabilized with decrease of hemolysis parameters, stable hemoglobin levels and reconstitution of kidney function after withdrawal of hemodialysis. At the time of discharge from hospital laboratory results were stabilized or even normalized: creatinine 2.04 mg/dl (180.34 μmol/l), bilirubin 0.5 mg/dl (8.55 μmol/l), LDH 207 U/L (3.45 μkat/l), INR 1.26, hemoglobin 7.6 g/dl (4.72 mmol/l). In a follow-up visit 4 months later kidney function was also normalized and the patient had returned to normal daily life.

## Discussion

**Fig. 3:**
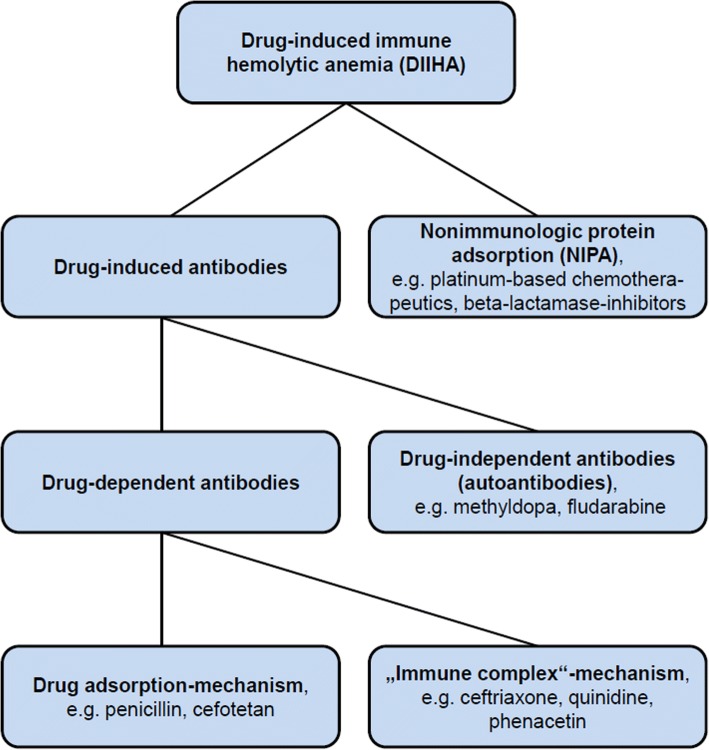
Systematic overview of different types of drug-induced immune hemolytic anemia

Drug-induced immune hemolysis is a rare (estimated incidence about 1/1,000,000/year), but potentially life-threatening complication and therefore an early and correct diagnosis is crucial [3, 6, 11]. Several mechanisms causing drug-induced hemolysis have been described during the last decades. Basically, it must be distinguished between *direct erythrocytotoxic* effects of drugs causing hemolysis, e.g. hemolysis by the antiviral drug ribavirin [12] and *drug-induced immunologic reactions* leading to extra- or intravascular hemolysis. The latter is a type of immune-hemolytic anemia (IHA) and called drug-induced immune hemolytic anemia (DIIHA). In general, DIIHA can be mediated through drug-induced antibodies or through a mechanism called nonimmunologic protein adsorption (NIPA), which is not triggered by antibodies [1, 11, 13]. Drug-induced antibodies can be subdivided into *drug-dependent* and *drug-independent* antibodies [1, 5, 11, 13]. *Drug-dependent* antibodies need the presence of the drug (or also of a drug-metabolite) to bind and lyse erythrocytes. In contrast, *drug-independent* antibodies can bind erythrocytes in absence of the causative drugs and are therefore true autoantibodies that can serologically not be distinguished from autoantibodies mediating warm autoimmune hemolytic anemia (WAIHA), so diagnosis relies on clinical response to cessation of the causative drug [1, 5, 6, 11, 13, 14]. It is considered that *drug-dependent* as well as *drug-independent* antibodies arise as an immunologic reaction against neoantigens formed by the binding of drugs to erythrocyte membranes. The drugs are haptens that need to be attached to a larger structure to become immunogenic [6, 11]. In case of DIIHA, this neoantigen consists of erythrocyte membrane and drug [1, 6, 11]. If the antibody recognizes only the molecular structure of the drug or a structure formed by membrane and drug together, it results in a *drug-dependent* antibody, that will only bind to erythrocytes and lead to hemolysis in the presence of the drug [1, 6]. In contrast, *drug-independent* autoantibodies are directed predominantly against a membrane structure and the drug is only a small and negligible part of the binding site. In this case, the antibody is able to bind erythrocytes also in the absence of the drug [1, 3]. *Drug-dependent* and *drug-independent* antibodies can be induced in the same individual during the same anti-drug reaction, supposing that they were generated by the same underlying mechanism [1]. Concerning drug-dependent antibodies, a further distinction can be made considering the binding mechanism of the drug to the erythrocyte: a covalent binding will result in a so-called “*drug-adsorption mechanism*” or “*penicillin-type*” reaction, while a rather loose binding will result in a so-called “*immune complex-type*” reaction, the latter being associated with a worse outcome due to formation of IgM-antibodies, complement activation and intravascular hemolysis (reviewed in [1, 3, 11]). Ceftriaxone-induced IHA is characterized by “*immune complex-type*” reactions and in a recent case of ceftriaxone-induced IHA antibodies with Rh specificity were described, that persisted 8 months after the drug reaction [15]. DIIHA by NIPA does not depend on any drug-induced antibody. NIPA is caused by some drug-induced, nonimmunologic modification of erythrocyte membranes, allowing the unspecific binding of diverse plasma proteins including IgG and complement factor 3 (C3), which leads to extravasal hemolysis in spleen [1, 5, 11]. Furthermore, some drugs can induce DIIHA by different mechanisms, e.g. platinum-based chemotherapies cause DIIHA by NIPA as well as drug-dependent antibodies by “*immune complex*”-mechanism [16]. An overview of the different mechanisms of drug induced hemolysis is depicted in Fig. 3.

Massive hemolysis and decrease in hemoglobin level are typical for ceftriaxone-induced IHA. *Mayer* et al. reported 12 cases of ceftriaxone-induced IHA with the nadir hemoglobin < 8 g/dl (4.96 mmol/l) in 9 cases and in 3 of these cases the nadir was even below 3 g/dl 1.86 mmol/l) [6]. *Arndt* et al. analyzed 25 cases of ceftriaxone-induced IHA including 17 children [2]. Ceftriaxone-induced IHA seems to be more frequent and more severe in children [2, 3, 6, 7, 11]. In the series of *Arndt* et al., 16 patients had a nadir hemoglobin < 5 g/dl (3.1 mmol/l), and among these 16 patients were 13 children. In three patients, the nadir was even < 1 g/dl (0.62 mmol/l) and all of them were children [2]. Children suffering from serious underlying diseases like HIV infection or sickle cell disease seem to be predisposed to develop ceftriaxone-induced IHA [17], and in sickle cell disease ceftriaxone-dependent antibodies may also lead to fatal sickle cell-crisis [18]. In our patient, the second hemolytic episode was much worse than the first one. This finding is typical for DIIHA [7, 11] and is due to a secondary immune response. The immune system of patients receiving a drug for the first time in their life needs some days to produce drug-dependent or also drug-independent antibodies [19]. In a review of 37 cases of ceftriaxone-induced IHA a weaker and self-limiting hemolytic episode associated with earlier ceftriaxone-application could be observed in 32% of these patients [7], underlining the assumption that especially secondary immune responses are responsible for severe DIIHA in general. A massive drop in hemoglobin levels in these patients led to severe complications such as shock, circulatory arrest, organ ischemia, disseminated intravascular coagulation (DIC), acute respiratory distress syndrome (ARDS) in 27 patients and 30% of the patients died [7]. Surprisingly, drug-dependent antibodies were detected also in healthy persons (blood donors/random patients) in much lower titers than in patients who developed DIIHA. This interesting finding might be associated with antibiotic use in industrial animal breeding [4, 5, 13], but the clinical relevance of this phenomenon is still unknown. However, one could speculate that these persons might be predestinated to develop clinically relevant antibody-titers and subsequent hemolysis after receiving therapeutic doses of the respective antibiotic [3]. The high prevalence of acute renal failure in patients with DIIHA in general is not only because of hypoperfusion/ischemia due to hemoglobin decrease and shock, but especially because of the nephrotoxicity of free hemoglobin and hemin [20]. Beyond their nephrotoxicity there are other proinflammatory effects of free hemoglobin and hemin that have to be considered in patients with DIIHA and might probably aggravate the clinical course of the patients (reviewed in [21]).

It has been noticed that ceftriaxone causes more severe clinical courses and more fatal outcomes than other drugs responsible for DIIHA [3, 6]. Ceftriaxone has been shown to induce primarily antibodies of IgM-type with accompanying IgG-antibodies [1–3]. IgM-type drug-dependent antibodies lead to binding and activation of complement, which results in intravascular hemolysis. In fact, intravascular hemolysis through complement-mediated lysis is a hallmark of “immune-complex-type” DIIHA [1, 11]. In line with this, Coombs’ direct antiglobulin test (DCT) in ceftriaxone-induced IHA is usually positive for C3 and, in some cases, also for IgG [1–3, 22–24]. However, negative DCT has also been described in ceftriaxone-induced IHA, probably because of massive hemolysis and therefore lack of intact complement−/antibody-loaded erythrocytes in this special case [25]. In our patient a positive DCT was observed for IgM, IgG and C3d.

Most importantly, if DIIHA is suspected, the suspicious drug must be stopped immediately. Discontinuation of the drug is the most important treatment measure concerning the patient’s outcome. In children with ceftriaxone-induced IHA, 8 of 9 patients, whose ceftriaxone therapy was stopped immediately, survived. In contrast, children without cessation of ceftriaxone treatment after diagnosis had a mortality of 50% [8]. DIIHA patients should be admitted to an intensive care unit to provide optimal supportive care and if required circulatory support. Transfusion of red blood cells will be done to the necessary amount. Recently, a case of ceftriaxone-induced IHA was reported with a patient refusing transfusions for religious reasons (Jehova’s witness). In this case the patient could be stabilized with daily application of erythropoietin, ferrous sulfate, folic acid and vitamin B12 [26]. In many cases, patients are given steroids. However, there is no proven benefit and therefore no recommendation for steroid therapy in DIIHA, at least as far as *drug-dependent* antibodies are involved [3, 11, 14]. In general, reports of successful use of steroids in DIIHA are usually confounded by the withdrawal of the responsible drug at the same time [3, 11]. In cases of *drug-independent* antibodies, which are autoantibodies, steroid therapy can be tried [3, 14], but also in these cases, the immediate withdrawal of the responsible drug is the most important therapeutic measure in order to stop the immunologic stimulation. Additionally, in cases of *drug-independent* antibodies, intravenous immunoglobulins (IVIG) can be given, if there is evidence of intravascular hemolysis, like in treatment of WAIHA [27]. Administration of high-dose IVIG has been successfully used in a child with severe ceftriaxone-induced IHA and a nadir hemoglobin of 2.2 g/dl (1.37 mmol/l) [24]. However, the question remains open whether the positive outcome of the patient was due to IVIG therapy or due to cessation of ceftriaxone. In some cases, plasmapheresis/plasma exchange has been used for treating DIIHA [3, 7, 8]. It could be speculated that removing drug-induced antibodies from the patient’s serum actively via plasmapheresis could be helpful in patients with “*drug adsorption-type*” DIIHA or with severe renal failure, where the causative drug is not eliminated within its normal half-time and might therefore trigger a prolonged hemolysis as well as an intensified immunologic stimulation.

As DIIHA of “*immune complex-type*” is due to complement-mediated intravascular hemolysis, one is tempted to speculate that a therapy with eculizumab, a complement inhibitor which hinders the formation of the “*membrane attack complex*”, could be helpful in these patients. Eculizumab is successfully used in paroxysmal nocturnal hemoglobinuria and (atypical) hemolytic uremic syndrome, and there have also been reports of its use in autoimmune hemolytic anemia [28, 29]. To our knowledge, there is no report of the use of eculizumab in a patient with DIIHA to date. However, complement inhibitors might be an effective therapeutic option especially in cases with severe intravascular hemolysis [30].

After the diagnosis of DIIHA, there is an absolute contraindication for re-exposure of the responsible drug for the patient’s lifetime. The application of drugs of the same substance class should be considered very carefully, as there could be interactions of the *drug-depending* antibody and these similar drugs. In case of ceftriaxone-dependent antibodies e.g., cross-reactivity has been shown with cefotaxime [6, 11, 23], cefpodoxime proxetil [23], with cefamandole [11] and with cefoperazone [11]. In addition, *drug-dependent* antibodies are not necessarily directed against the drug itself, but can also be directed against a drug metabolite or against both the intact drug and its metabolite(s) [3, 19, 23], which makes crossreactions to drugs of the same substance class even more probable.

Antagonizing the toxic effects of free hemoglobin and free hemin could be an effective therapeutic strategy in future to prevent renal failure. In animal models of hemolysis, the application of haptoglobin (binding free hemoglobin) as well as of hemopexin (binding free hemin) has proven beneficial [31, 32], so maybe purified haptoglobin or hemopexin might become effective therapeutic agents for DIIHA one day.

## Conclusions

Our case demonstrates the severity of ceftriaxone-induced immune hemolytic anemia, a rare, but immediately life-threatening condition of a frequently used antibiotic in clinical practice. For the last decades, ceftriaxone has been one of the most common drugs responsible for DIIHA and has been associated with particularly severe outcome. In cases of unclear hemolysis, treating physicians should be aware of DIIHA and check the patient’s medication carefully. Suspected drugs have to be stopped immediately in order to prevent severe complications and fatal outcomes.

## Abbreviations

ARDS: Acute respiratory distress syndrome
CRP: C-reactive protein
DAT: Direct antiglobulin test
DIC: Disseminated intravascular coagulation
DIIHA: Drug induced immune hemolytic anemia
IgG: Immunoglobulin G
IgM: Immunoglobulin M
IHA: Immune hemolytic anemia
INR: International normalized ratio
IVIG: Intravenous immunoglobulins
LDH: Lactate dehydrogenase
NIPA: Nonimmunologic protein adsorption
PCT: Procalcitonin
WAIHA: Warm autoimmune hemolytic anemia
